# Investigation of Post Vaccination Reactions of Two Live Attenuated Vaccines against Lumpy Skin Disease of Cattle

**DOI:** 10.3390/vaccines9060621

**Published:** 2021-06-08

**Authors:** Zahra Bamouh, Jihane Hamdi, Siham Fellahi, Slimane Khayi, Mohammed Jazouli, Khalid Omari Tadlaoui, Ouafaa Fassi Fihri, Eeva Tuppurainen, Mehdi Elharrak

**Affiliations:** 1 MCI Santé Animale, Mohammedia 28810, Morocco; jihaneh.hamdi@gmail.com (J.H.); jazouli_med@yahoo.fr (M.J.); k.tadlaoui@mci-santeanimale.com (K.O.T.); m.elharrak@mci-santeanimale.com (M.E.); 2 Institut Agronomique et Vétérinaire Hassan II, B.P 6202, Rabat 10112, Morocco; s.fellahi@iav.ac.ma (S.F.); o.fassifihri@iav.ac.ma (O.F.F.); 3 CRRA-Rabat, National Institute for Agricultural Research (INRA), Rabat 10101, Morocco; slimane.khayi@inra.ma; 4 Institut für Internationale Tiergesundheit/One Health, Friedrich-Loeffler-Institut Federal Research Institute for Animal Health, 10 17493 Greifswald-Insel Riems, Germany; eeva.tuppurainen@fli.de

**Keywords:** lumpy skin disease virus, safety, Neethling disease, vaccination, cattle

## Abstract

Lumpy skin disease virus (LSDV) causes an economically important disease in cattle. The only method for successful control is early diagnosis and efficient vaccination. Adverse effects of vaccination such as local inflammation at the injection site and localized or generalized skin lesions in some vaccinated animals have been reported with live vaccines. The aim of this work was to compare the safety of two lumpy skin disease (LSD) vaccine strains, Kenyan (Kn) Sheep and Goat Pox (KSGP O-240) and LSDV Neethling (Nt) strain, and to determine the etiology of the post-vaccination (pv) reactions observed in cattle. Experimental cattle were vaccinated under controlled conditions with Nt- and KSGP O-240-based vaccines, using two different doses, and animals were observed for 3 months for any adverse reactions. Three out of 45 cattle vaccinated with LSDV Nt strain (6.7%) and three out of 24 cattle vaccinated with Kn strain (12.5%) presented LSD-like skin nodules, providing evidence that the post-vaccination lesions may not be strain-dependent. Lesions appeared 1–3 weeks after vaccination and were localized in the neck or covering the whole body. Animals recovered after 3 weeks. There is a positive correlation between the vaccine dose and the appearance of skin lesions in vaccinated animals; at the 105 dose, 12% of the animals reacted versus 3.7% at the 104 dose. Both strains induced solid immunity when protection was measured by neutralizing antibody seroconversion.

## 1. Introduction

Lumpy skin disease (LSD) is an economically devastating infectious disease of cattle occurring in Africa, the Middle East, southeastern Europe, northern Caucasus, the Russian Federation, the Indian subcontinent, and south, east, and southeast Asia with a high economic importance [1]. Lumpy skin disease is caused by the LSD virus (LSDV), belonging to the *Poxviridae* family, genus *Capripoxvirus* [2]. Transmission is due to arthropod vectors, including biting flies, mosquitoes, and hard ticks [3,4]. Although direct transmission is not considered a major route of transmission during epizootics, it may occur by contact through contaminated saliva, semen, milk, or skin lesions [4,5,6,7,8,9]. Lumpy skin disease infections in cattle range from inapparent to severe manifestations, producing clinical symptoms such as fever associated with a marked reduction in milk yield, ocular and nasal discharge, hypersalivation, and enlarged lymph nodes. Lumpy skin disease is characterized by cutaneous nodules of 2–5 cm in diameter across different body locations that evolve into large nodules that may become necrotic. Ulcers can be detected in the digestive and respiratory tracts, and pregnant cows may abort [10,11,12,13,14].

The most effective way to control LSD in endemic countries is to carry out large-scale vaccinations with safe and effective vaccines. All three members of the *Capripox* genus have been used to immunize cattle against LSD. The homologous South African Neethling (Nt) strain has been used worldwide, while Kenyan (Kn) strain use is limited to a few countries: Israel [15], Ethiopia [16], and Egypt [17]. Heterologous strains based on sheep pox (RM65 and Romania strains) or goat pox (Gorgan strain) are also used to prevent LSD, but the efficacy of the different vaccine products varies [16,18,19]. Lumpy skin disease virus-based vaccines may induce adverse reactions such as fever and lymphadenitis. Neethling disease was reported by Yerhun et al. with Nt vaccines [15] and by Abutarbush et al. with 10 sheep doses of RM65 vaccine [20]. The percentage of vaccinated animals showing LSD-like nodules after vaccination ranges from 0.38% to 12% according to field observations [21,22]. According to Israeli experience, the LSD Nt vaccine caused only mild adverse effects at a very low incidence (0.38–0.6%) [21]. In Croatia, adverse reactions were reported in 0.09% of the vaccinated animals using a homologous vaccine [23]. In a study carried out by Greek scientists, a local reaction at the vaccination site was detected in 12% and generalized small-sized lumps in 9% of animals vaccinated with a commercially available LSD Nt strain vaccine [22]. LSD-like lesions, following vaccination, were reported by researchers in Jordan in 8% of cattle vaccinated with RM-65 vaccine strain [24]. The differences in reported percentages of LSD-like nodules post vaccination are probably associated with differences in pre-existing immunity such as previous vaccinations or with the reluctance of farmers to report the side-effects in their vaccinated herds. The cause for the appearance of the post-vaccination skin nodules may vary across vaccine strains, vaccine dosage used, immune status of the vaccinated animal, or recurrent infections of the animals.

To investigate the side-effects caused by two different attenuated homologous LSDV strains (Nt and Kn KSGP-O 240), four groups of cattle were vaccinated in a controlled environment, using two different doses. The vaccinated experimental animals were closely monitored for appearance of the side-effects, and seroconversion was evaluated for 3 months post vaccination.

## 2. Materials and Methods

### 2.1. Vaccine Preparation

The attenuated Nt LSD strain, commonly used in vaccine preparation, was provided by the Pirbright Institute. The passage number of this strain is not known. To prepare the vaccine, the strain was once propagated in ovine primary testis cells in our laboratory and then harvested. A stabilizer (4% peptone, 8% sucrose, and 2% glutamate) was added to the virus suspension, which was then freeze-dried. The vaccine was tested for bacterial sterility, strain identity, purity, and infectious titer. Experimental animals were vaccinated with a volume of 2 mL of 10^4.0^ TCID_50_ (low dose) or 2 mL of 10^5.0^ TCID_50_ (high dose). 

The LSD Kn (KSGP O-240) strain was also provided by the Pirbright Institute and used to prepare the other live attenuated vaccine of this study. The strain was passed three times in primary lamb testis cells and presented a titer of 10^5.0^ TCID_50_/_mL_; the vaccine was then injected subcutaneously ito five naïve cattle (5 mL each). Any animal presenting skin lesions post vaccination was submitted to viral isolation and PCR. Re-isolation was performed in ovine primary testis cells by inoculation of the homogenate of the skin inflammation tissue. The harvested virus was tested for identity and purity and used to prepare the vaccine as described above for the Nt strain. Experimental animals were vaccinated using a volume of 2 mL of 10^4.0^ TCID_50_ (low dose) and 10^5.0^ TCID_50_ (high dose). 

LSD Nt and Kn vaccines were reconstituted with a sterile phosphate saline diluent. Directly after the reconstitution, the vaccines were inoculated subcutaneously into the neck region of the experimental cattle.

### 2.2. Sequencing

The whole-genome sequencing of the LSD Nt strain was performed by Sciensano, Belgium to confirm the identity of the strain. The LSDV Kn strain DNA was obtained from a skin sample collected from a calf used to refresh the strain. The DNA was extracted using an Isolate II Genomic DNA kit (Qiagen, Germany) according to the manufacturer’s instructions. The whole-genome sequencing was carried out by the Eurofins Genomics company using Genome Sequencer Illumina HiSeq (Constance, Germany) and sequence mode NovaSeq 6000 S2 PE150 XP platforms. The raw reads were trimmed using CLC genomics workbench v12 (Qiagen) (limit = 0.05, ambiguous nucleotides *n* ≤ 2). The trimmed reads were de novo assembled using CLC genomics workbench v12. 

Full-length GPCR and RPO30 genes were retrieved from the assembly. The resulting sequences were aligned together with the reference sequences retrieved from GenBank for each gene using the CLUSTALW algorithm [25] in BioEdit 7.2.5 [26]. Molecular phylogenetic analyses were performed using MEGA 10.2.2 [27]. The evolutionary history was inferred using the neighbor-joining method [28], and confidence on branching was assessed using bootstrap resampling (1000 replicates) [29]. The evolutionary distances were computed using the Kimura two-parameter method [30]. 

The purpose of the sequencing was to confirm identity with the reference strain (ID: KX683219) reported by Vandenbussche et al. 2016 [31] and analyze variants of the obtained sequence (indels and single-nucleotide polymorphisms (SNPs)) [31].

### 2.3. Experimental Animals

The study protocol was approved by the Internal Laboratory Ethic Committee of MCI Santé Animale (Protocol number RD1V1730). In addition, the international guidelines for caring and handling of experimental animals as described in Chapter 7.8 of the Terrestrial Animal Health Code and Directive 2010/63/UE of the European Commission [32,33] were followed. 

Seventy-three Holstein-cross breed, healthy calves, 4 to 6 months of age, were purchased from a selected cattle breeding farm. Currently, Morocco is free of LSD, and the experimental cattle were housed in fly-proof pens at cattle quarantine facilities located in the northwest region of Morocco. Prior to the onset of the experiment, the animals were allowed to acclimatize for 15 days and were monitored daily for fever and appearance of any nonspecific clinical signs. Blood samples were collected prior to vaccination to confirm the absence of anti-LSD antibodies using the virus neutralization test (VNT). Cattle were fed a complete balanced diet and water ad libitum during acclimatization and experimental periods. Cattle were randomly selected and were divided into five homogenous groups: G1: (15 animals) vaccinated with 2 mL of 10^4^ TCID_50_/_mL_, the low dose of Nt vaccine, G2: (30 animals) vaccinated with 10^5^ TCID_50_/_mL_, the high dose of Nt vaccine, G3: (12 animals) vaccinated with 10^4^ TCID_50_/_mL_, the low dose of Kn vaccine, G4: (12 animals) vaccinated with 10^5^ TCID_50_/_mL_, the high dose of Kn vaccine, and G5: (four animals) unvaccinated control animals inoculated with a placebo solution. Cattle were housed in separate boxes at a level 3 biosecurity containment and injected subcutaneously on the right side of the neck with 2 mL of the appropriate vaccine and observed for 90 days post vaccination (pv). The body temperature was recorded daily for 14 days, and general body condition was evaluated and scored for each animal. Clinical scoring for each parameter ranged from 0–2 or 0–5 as outlined in Table 1. To preserve the trial objectivity, animal carers or investigators were blinded to the vaccine type and dose. Roles and responsibilities were prespecified. Clinical signs were scored as described in Table 1; a total cumulative score of assessed signs per animal and group per day was calculated. Blood samples were collected from each animal by jugular venipuncture on day (D) 0 (before vaccination), D7, D14, D21, D28, D35, D42 D60, and D90 pv. Sera were stored at −20 °C until analysis. At the end of the experiment, cattle were euthanized using humane slaughter.

### 2.4. Virus Neutralization (VNT)

The humoral response of animals to LSD vaccination was evaluated by testing serum samples using the VNT method as described in the OIE current version of the Terrestrial Manual (OIE Chapters 2.7.12). Sera were heat-inactivated, and serial 1:3 dilutions were mixed with a constant dose of LSD virus followed by incubation for 1 h. A Madin-Darby Bovine Kidney (ATCC^®^ CCL-22™, Manassas, VA, USA) cell suspension was then added, inoculated on cells, and observed after 7 days for the presence of cytopathic effect (CPE). To validate the used viral dose, dilutions without sera were inoculated on cells and observed for 7 days for the presence of CPE. The neutralizing antibody titer was calculated according to the method used by Reed and Muench (1937).

### 2.5. PCR

Nasal and oral swabs were collected from vaccinated cattle on days 3, 6, 9, 12, 21, 28, and 35 pv to detect LSDV excretion using quantitative real-time PCR (qPCR). All samples were transferred to the laboratory on ice. Swabs were collected in 2 mL of phosphate-buffered saline (PBS) and then centrifuged at 2000 rpm for 20 min at 4 °C degrees. Viral DNA was extracted using ISOLATE II Genomic DNA Kit (Bioline, London, UK). Samples were tested by a qPCR TaqMan assay that detects the 89 bp region from ORF074 encoding the intracellular mature virion envelope protein P32 within SPPV, GTPV, and LSDV. Real-time PCR was conducted with a Luna Universal Probe QPCR Master Mix Kit (New England Biolabs, Ipswich, USA). The reaction was performed in 96-well optical reaction plates, containing 12.5 µL of 2× Universal Probe QPCR Master Mix, 1 µL of each primer cited by Bowden [6], 5 µL of template (DNA), and 5 µL of nuclease-free water. The qPCR assay was run and optimized in ABI7500 (Applied Biosystems, Foster City, California, USA) with the following cycling conditions: 95 °C for 10 min, followed by 45 cycles of PCR at 95 °C for 15 s and 60 °C for 1 min.

**Virus recovery from cutaneous lesions:** The LSD virus was reisolated from the skin sample collected from the only animal that showed local inflammation of 3 to 5 cm of diameter at the injection site. Identity of the agent was confirmed by the development of typical CPE followed by cell lysis at 5 days post infection and through PCR confirmation.

**Statistical analyses** were performed using Student *t*-test models. Comparisons were carried out between vaccinated groups on the basis of clinical scoring and Neethling-like disease presence. Values of *p* ≤ 0.05 were considered significant.

**GenBank accession numbers.** The full genome sequences of LSD Kn KSGP O-240 strain after refreshment on cattle were analyzed in this study and deposited in GenBank (accession number MW631933).

The sequences of RPO30 and GPCR genes of LSD Kn strain under study were compared with the sequences of LSD already available on the public sequence databases of the GenBank under the following accession numbers for the RPO30 gene: KJ818288; MT448696; MT448695; MT448694; MT448693; MT448692; MT448691; MN967006; MT228884; MT228883; MT228882; MT228881; MG201832; MK302094; GU119944; KJ818290; KJ818289; KJ818288; MN518933; MN598007; GU119938; GU119943; GU119945; GU119946; GU119947; GU119948; GU119951; GU119952; GU119950; GU119937; KX683219.

Comparisons were also made using the following accession numbers for the GPCR gene: FJ869367; FJ869372; FJ869368; MT448700; FJ869352; FJ869369; FJ869370; FJ869373; FJ869371; KP663706; FJ869377; MT448701; FJ869374; FJ869375; MK452255; FJ869366; MT448699; MT448697; MT448698; FJ818283; FJ818282; FJ818281; KX683219; MN508357; MK358808; FJ869376; MN598006; FJ869365; MF156212; MG970343.

## 3. Results

Virus isolation: Both Nt and Kn LSD strains grew on ovine primary testis cells. Strains showed characteristic CPE starting from day 3 and were harvested on day 5 pi. The titration performed on both viruses revealed a titer of 10^6.9^ and 10^7.0^ TCID_50_/_mL_ for Nt and Kn viruses, respectively. Final vaccines complied with analytical QC testing [34].

The LSD Nt strain used in this experiment showed 100% similarity with sequence data from LSDN stored in the GenBank (ID: AF409138). The whole-genome sequence and assembly of KSGP O-240 strain refreshed on experimental cattle contained 146,090 bps and 26% G + C content. Variant calling, using the reference sequence (KX683219), revealed seven variants: two single-nucleotide deletions, three single-nucleotide substitutions (SNVs), and two single-nucleotide insertions (Table 2).

Despite those seven noted changes in the KSGP O-240 strain, only three genes were affected with no consequences for strain identity. The Kn strain used shared 99% homology with the LSD strain (ID: KX683219). According to the complete genome sequence, the strain KSGP O-240 was confirmed to be an LSDV.

**Figure 1:**
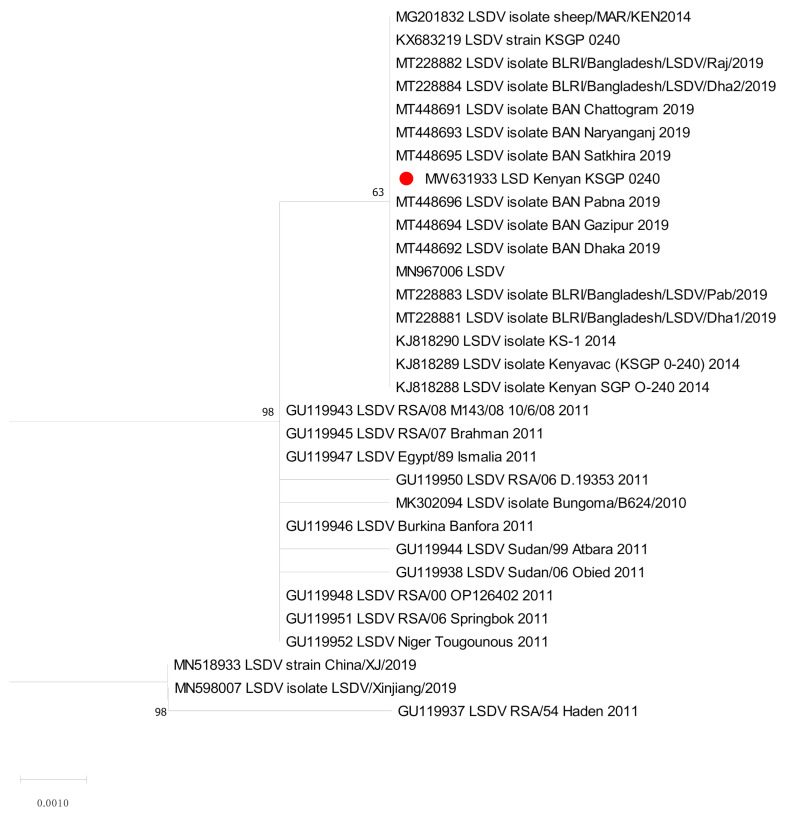
Molecular phylogenetic analysis of the *Capripoxvirus* RNA polymerase subunit gene (RPO30). The RPO30 sequence obtained in this study was marked with red circle shaped symbols.

**Figure 2:**
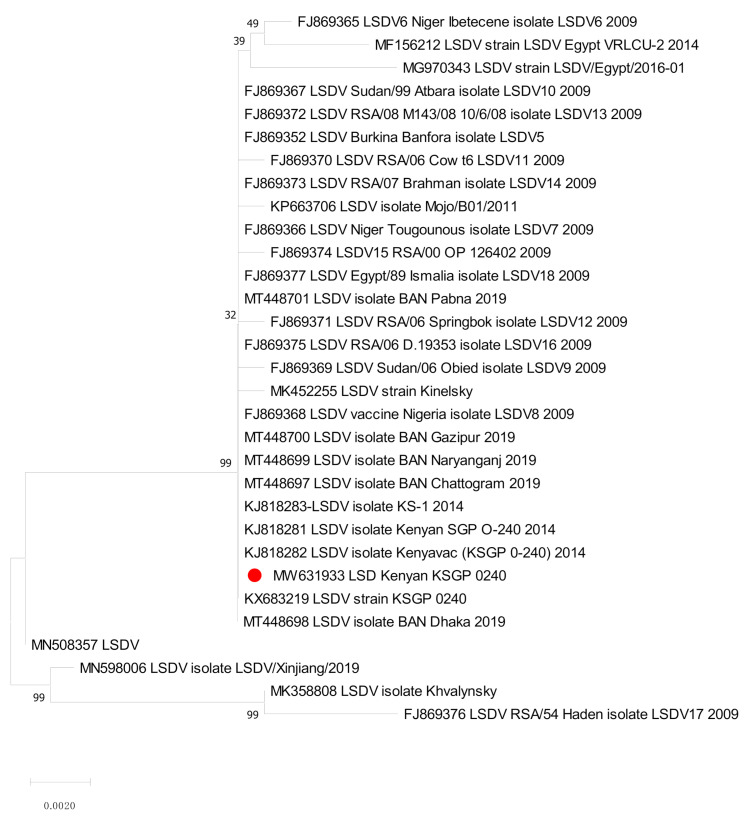
Molecular phylogenetic analysis of KSGP O-240 strain based on the *Capripoxvirus* G-protein-coupled chemokine receptor (GPCR) gene. The GPCR sequence obtained in this study was marked with red circle shaped symbols.

The sequences of RPO30 and GPCR genes were determined for the Kn strain before and after refreshment. These were compared with the sequences of LSDV already available on the public sequence databases. Molecular phylogenetic analyses were performed on the coding regions of the RPO30 gene (31 sequences, 606 positions; Figure 1) and the GPCR gene (30 sequences, 1146 positions; Figure 2). These results confirmed the identification made using real-time PCR.

The phylogenetic analysis of the RPO30 and GPCR genes, with multiple sequence alignments, revealed that our Kn strain was closely related to LSDV KSGP O-240, LSDV Kn, and Bangladesh LSD isolates.

On the RPO30 tree (Figure 1), the Kn strain in this study clustered with Bangladesh isolates 2019, LSDV KSGP O-240 (KJ818288) known as LSDV KS1, and LSDV KSGP O-240 (KX683219). On the GPCR tree (Figure 2), our strain was closely clustered with LSDV KSGPO-240 (KX683219) and LSDV KSGP-0240 (KJ818281) and (KJ818282).

**Figure 3:**
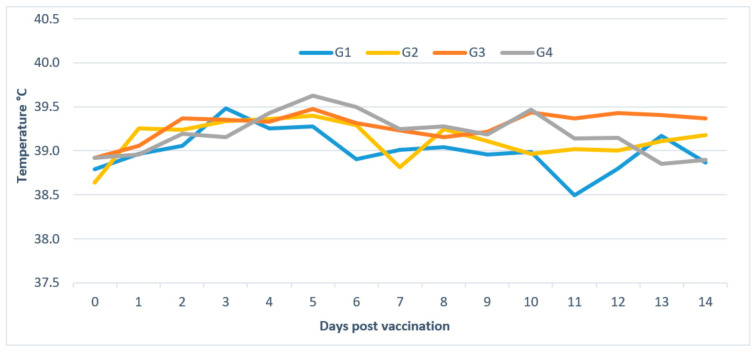
Average temperature of animals of G1 vaccinated with low dose of Neethling strain, G2 vaccinated with high dose of Neethling strain, G3 vaccinated with low dose of Kenyan strain, and G4 vaccinated with high dose of Kenya strain.

**Body temperature:** In Group 1, out of 15 animals, seven presented moderate hyperthermia (maximum temperature recorded was 40 °C) for 2 days, starting from D2 (average duration of 0.93 days/animal). In Group 2, 13 animals among 30 developed moderate hyperthermia (maximum temperature recorded is 40.8 °C), two animals for 1 day, five animals for 3 days, three animals for 5 days, and three animals for 8 days (1.87 days/animal). In Group 3, five calves among 12 showed hyperthermia (maximum temperature recorded is 41.9 °C), two animals for 5 days and three animals for 6 days (2.33 days/animal). In Group 4, nine among 12 animals developed hyperthermia (maximum temperature recorded is 41.1 °C), two animals for 1 day, two for 6 days, four for 3 days, and one for 9 days (2.92 days/animal). The body temperature of unvaccinated cattle (G5) remained normal and did not exceed 39 °C (Figure 3).

**Clinical scoring:** Cattle vaccinated with Nt or Kn strains remained healthy; only cattle of G4 presented a clinical scoring of 2.5, due to the appearance of LSD-like lesions. The control group did not show any clinical signs (Table 3). Six out of 69 (8.7%) vaccinated animals showed LSD-like nodules in different parts of the body. In G1, one Neethling disease case was observed, whereas two cases and three cases were observed in G2 and G4, respectively. No cases were noted in G3 (Table 3).

Briefly, in G1 vaccinated with Nt low dose, one animal out of 15 (6.7%) cattle showed cutaneous nodules localized in both sides of the neck measuring 0.5–1 cm, from day 14 pv. In G2, one animal showed lesions in the neck of the same size from day 14 pv, and another showed head and neck nodules of 1–2 cm of diameter from D13 pv (6.7%). In two animals of G2, local inflammation reactions of 3 × 3 cm in diameter were detected at the inoculation sites, starting between days 7 and 9 pv (Table 4).

**Figure 4:**
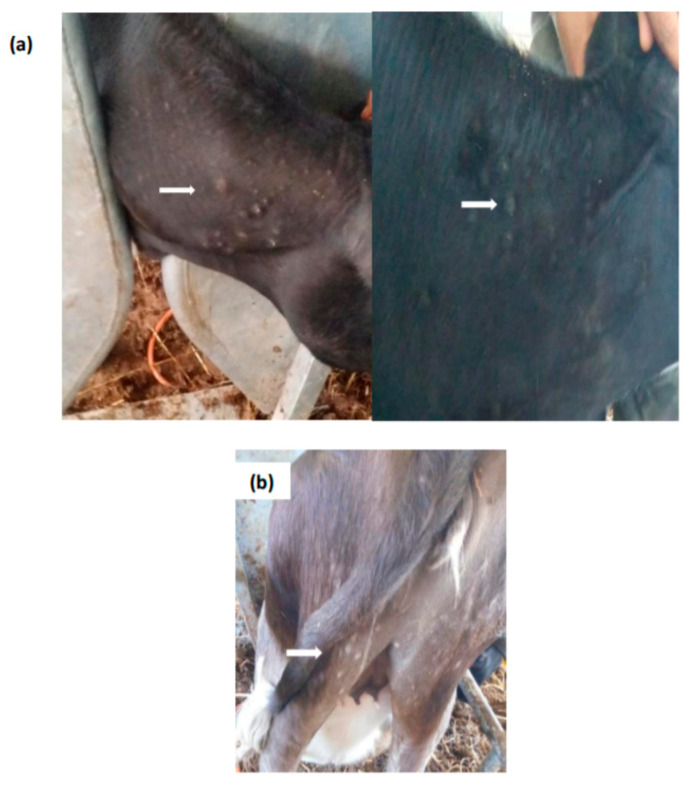
(**a**) Localized skin lesions in the neck seen in cattle vaccinated with Neethling strain (G2). (**b**) Localized skin lesions in thighs regions and perineum seen in cattle vaccinated with Kenya strain (G4). Arrows show skin lesions.

In G4, which comprised a total of 12 cattle vaccinated with LSD Kn high dose, the percentage of animals showing generalized skin lesions after vaccination was as high as 25%. One animal displayed generalized nodules across the whole body, starting from D13 pv. The second animal showed skin nodules of 0.5 × 2 cm in diameter which appeared 21 days pv in the neck, posterior parts of the body, and perineum (Figure 4). In the third calf, small lesions 0.5–1 cm in diameter were noted on day 7 pv in the head and perineal area; on day 14 pv, skin lesions become more accentuated in the head and thigh regions with nodules detectable by palpation in the neck and chest. A local reaction of 2–3 cm in diameter was also observed at the inoculation site in this animal. 

Observed nodules in these animals were located on various parts of the body but mainly on the head and neck. The skin lesions looked like LSD nodules but were smaller and led to crusts before healing. Animals stayed healthy, with good bodily condition with no other clinical signs observed. The cutaneous lesions disappeared in 3 weeks and all affected cattle healed in about 1 month.

Absence of skin lesions in unvaccinated animals of G5 confirmed the non-diffusion of the vaccine strain under Biosafety Level 3 (BSL3) conditions in a controlled environment.

**Seroconversion:** Animals vaccinated with LSD Nt at low dose (G1) showed seroconversion starting on day 14 pv (20%) and reached 47% by day 42 pv. Those cattle vaccinated with the LSD Nt high dose (G2) showed a positive antibody response starting on day 7 pv and reached a percentage of 73% on day 35 pv. Regarding the LSD Kn vaccine at low dose (G3), animals seroconverted from day 14 pv and reached 100% on day 21 pv. For cattle vaccinated with high dose (G4), 25% seroconverted already on day 7 pv and all of the animals were seropositive by day 21 pv (Table 5). All animals remained positive for more than 3 months. The average neutralizing antibody titers recorded on D28 post vaccination was 1.2 at low dose (G1) and 1.7 at high dose (G2) for Nt strain and 1.6 at low dose (G3) and 2.1 at high dose (G4) for Kn strain.

Absence of seroconversion in animals of G5 confirmed the non-diffusion of the vaccine strain.

### 3.1. Viral Excretion 

No viral DNA was detected in oral or nasal swabs collected from cattle vaccinated with Nt and Kn strains, except in samples collected from those six animals that showed a Neethling-like disease after vaccination. In cattle showing severe vaccine side-effects, the virus was detected in oral and nasal swabs first on day 9 pv, and they remained PCR-positive until day 28 pv, with a cycle threshold (C_t_) value ranging between 26.7 and 36.0. On day 35 pv, all six animals tested negative again. Skin crusts taken from animals showing skin lesions tested qPCR-positive with the C_t_ varying between 19.1 and 36.0 on day 23 pv. Virus isolation was attempted from a sample collected 23 days pv and showed characteristic CPE on cells, indicating that the virus was live. However, for this sample, no sequencing of the DNA of the isolated virus was carried out. 

All unvaccinated animals remained clinically healthy and PCR-negative throughout the trial.

### 3.2. Statistical Analysis 

The Neethling-like disease parameter was significantly higher (*p* ≤ 0.05) in G4 of cattle vaccinated with LSD Kn vaccine at high dose (25%) compared to cattle vaccinated at low dose (0%). Clinical scores were significantly higher (*p* ≤ 0.05) in animals vaccinated with LSD Kn strain compared to animals vaccinated with LSD Nt strain. Subject to the unequal number of animals in each group, the average clinical score for cattle vaccinated with Kn vaccine was 1.75, whereas the clinical score was 1 in cattle vaccinated with LSD Nt vaccine. 

## 4. Discussion

Lumpy skin disease outbreaks cause substantial financial losses for farmers and the whole cattle farming industry in endemic and newly affected regions [1]. The most effective tool to limit spread of the vector-borne LSDV is to carry out large-scale vaccination campaigns, comprising the whole cattle population together with cattle movement restrictions. Live attenuated LSDV vaccines are known to provide a good protection in cattle [35].

Vaccination with the LSD Nt strain, using a high vaccination coverage allows an efficient control of the spread of the disease [36]. Heterologous vaccines based on sheeppox strain have been used for cattle against LSDV in some countries [37]. Protection by sheeppox RM-65 strain at 10 times dose of small ruminants has been reported to be partial for LSD [16,21,38,39,40]. Goatpox virus vaccines have been shown experimentally to be effective against LSD [7,41], but more data on the efficacy of these vaccine in the field need to be obtained.

The most common vaccine used against LSDV worldwide is the South African “Nt” strain [12,42]; as such, more animals were included in groups vaccinated with the Nt strain (G1 and G2) than in the Kn vaccine groups (G3 and G4). The number of experimental animals allowed the detection of LSD-like disease and comparison between Nt and Kn strains, as well as high and low vaccine doses. 

Live Nt strain-based vaccines have been associated with adverse vaccine reactions, raising concerns on the safety of vaccination [18,22,43,44]. In addition, no post-vaccination safety data are available for Kn strain in cattle, as this vaccine has been mainly used in small ruminants to prevent sheeppox and goatpox. Very few studies have reported vaccination of cattle with Kn SGP O-240 or O-180 strains against LSD [7,15,16].

The aim of the study was to investigate the etiology of the post-vaccination reactions observed in cattle. The most commonly noted side-effect is a small local reaction at the vaccination site, caused by local replication of the vaccine virus, which can also be considered as an indication of protection [45]. Some animals may show generalized LSD-like nodules which, in most cases, can be differentiated from those caused by the virulent LSD field strains, as they are smaller and disappear within 1–3 weeks [24,43]. Currently, there is no consensus on the proportion of animals showing vaccine side-effects and the real consequences of vaccination, considering cattle production, such as effect of vaccination on the milk yield.

In this study carried out in a controlled environment, the clinical reaction of naïve healthy calves after vaccination with Nt and Kn vaccines at two different dosage levels was investigated, namely, 10^4.0^ (low) and 10^5.0^ TCID_50_ (high). To our knowledge, this is the first study of this phenomenon, using a sufficient number of animals in a high containment facility. Within all groups, six experimental animals out of 69 (8.7%) showed a “Neethling disease” vaccine reaction, and only one of them, vaccinated using the high dose of Kn vaccine (G4), presented a generalized and severe post-vaccination reaction. The study demonstrated that, for LSD Nt and Kn vaccines, the appearance of vaccine induced LSD-like disease was not strictly related to a particular strain, as reaction was observed after vaccination with both strains. However, the percentage of LSD-like disease was higher in those groups (G4) vaccinated with the Kn strain. Cattle vaccinated with Nt strain showed three LSD-like cases among 45 animals (6.7%), while cattle vaccinated with the Kn strain revealed three cases among 24 (12.5%). Regarding vaccine dose, 3.7% of the animals presented LSD-like disease at the low dose, while 11.9% showed skin lesions when vaccinated with the high vaccine dose, proving a clear dose–effect relationship, more visible with the Kn strain (25%) at a high dose versus none at the low dose. The size and the localization of LSD-like nodules were also more accentuated in calves vaccinated with the high vaccine doses. These results are compatible with a study that showed the presence of systemic vaccine adverse reactions only in cattle that received the high dosage of sheeppox RM65 vaccine [20]. All confirmed cases of Neethling disease appeared within a period of 7–17 days from vaccination, and animals recovered within 11–17 days.

However, nasal and saliva swabs and skin lesions collected from vaccinated animals showing LSD-like disease tested LSD-positive by PCR. However, further well-designed investigations on potential transmission of the vaccine virus to cohabitant animals under field conditions are required. In a study by Bedekovic and coworkers, the level of vaccine virus was higher in the skin lesions, which may affect spread via mechanical transmission by insects or ticks feeding on skin lesions [43]. Interestingly, in a Croatian study, when the vaccine virus was isolated and sequenced from the skin lesions of the vaccinated animals, the obtained data showed that the virus remained fully attenuated [46].

LSD-like skin lesions are often detected in vaccinated animals after vaccination campaigns in newly affected countries [23]. The high-production dairy cows seem to be most susceptible to the natural disease [47,48], and the same could be to be the case for the vaccine virus. Severe vaccine side-effects may hamper the eradication of LSD because of the concerns of transmission of the live vaccine virus, recombination of the vaccine with field strains, and unwillingness of farmers to use the vaccine. Previously, too low attenuation level of the vaccine strain, use of high vaccine dosage, or factors related to recurrent infections in vaccinated animals have been suggested to be the reason for the appearance of the vaccine side-effects [38]. In this study, the host-dependent factors could not be considered, because only calves in good health and condition were used. However, the Kn strain presented more “Neethling disease” cases, with a percentage of 25% at high dose (3/12), compared to 6.7% (2/30) with the Nt strain at the same dose. Moreover, a long duration of fever reaction was recorded, even when the Kn strain was used at a low dose. In previous studies, it was suggested that the low-level attenuation of KSGP O-240 vaccine is likely to be not sufficient for the safe use of this vaccine in cattle, causing clinical disease in vaccinated animals [38]. The Kn strain used in this study was refreshed by carrying out an additional passage in experimental calves to increase its replication capacity.

One of the parameters that correlates with the protection provided by the vaccine against LSDV is the humoral response in vaccinated animals. However, the absence of antibodies does not necessarily mean the absence of protection, because the cell-mediated immunity plays a dominant role in capripox infections [49]. Some animals may remain seronegative after natural infection or vaccination, even though they would be fully protected against the disease [14,45]. In this experiment, animals in group G2 that received the high dose of LSD Nt strain vaccine showed a better humoral response than those that received the low dose. Kn vaccines induced a better antibody response than Nt vaccines. In another study, a significant increase of capripoxvirus-specific antibody titers was seen from day 21 up to day 42 after vaccination, and antibodies remained detectable for about 7 months [50]. Hamdi and coworkers reported that the live vaccine induced an antibody response reaching 50% on day 28 pv [51].

In the study by Milovanović and coworkers, LSD-specific antibodies were detected with VNT for 46 to 47 weeks after vaccination in 35% of vaccinated cattle with the LSDV Nt vaccine (Onderstepoort Biological Products, OBP, Pretoria, South Africa) [50]. In another study, antibodies against LSDV were detected by VNT in 34% of samples for 20 days after vaccination using the OBP Lumpy Skin Disease vaccine (Onderstepoort Biological Products) [52]. However, in an Ethiopian study, vaccination of cattle with KSGP O-180 and Nt strains did not elicit a detectable antibody response using the indirect fluorescence antibody test (IFAT). All experimental animals vaccinated with the Gorgan goatpox strain were fully protected against a challenge with a highly virulent LSD field strain, although only 50% of them tested seropositive using the IFAT [7]. Absence of seroconversion in cattle vaccinated with Nt and KSGP O-180 strains was associated with poor immunogenicity of these locally produced vaccines, likely to be due to over-attenuation or some other failure in these vaccine products [7]. In the present study, the original Kn LSD strain was passaged 3–4 times at the Pirbright Institute, and one additional passage in cattle was carried out during this study. This is a very low passage number for the homologous vaccine against LSDV. Interestingly, during the refreshment passage prior to the preparation of the vaccine, the Kn strain caused a local reaction at the vaccination site only in one out of five inoculated calves. This finding can be explained by the low number of cattle used in the refreshment trial and is in agreement with the earlier observations on the appearance of clinical signs in experimental animals [5,7]. All animals vaccinated with the Kn strain (24/24) seroconverted, but a higher dosage of the Kn vaccine was less safe as 25% of vaccinated cattle showed LSD-like skin nodules after vaccination. Seroconversion was detected in all experimental animals vaccinated with the Nt strain.

A comparison of the sequence alignment of the GPCR and RPO30 genes showed that the Kn strain used in this study clustered at the nucleotide level with Kn vaccine strain LSDV KSGP O-240 and with recently analyzed LSDV Bangladesh isolates [53] A comparison of full genome sequences with KX683219 indicated the highest percentage sequence identity to the vaccine strain (99%).

The existence of vaccine-like field isolates with mixed characteristics between common field viruses and the vaccine used against LSD has been reported by Russian scientists [54]. More recently, a field LSDV isolated from Kurgan, Russia, exhibited similarities to LSDV KSGP O-240 and LSDV NI2490, according to an analysis of GPCR and RPO30 gene fragments [55]. The comparison of the GPCR gene indicated that the Kn strain used in our study is identical to the other KSGP O-240 strain and those LSDV strains currently circulating in Bangladesh [48]. Previously, it was suggested that KSGP O-240 strain vaccines are known to cause side-effects in cattle if used in naïve animals due to insufficient attenuation [38]. The similarity of the Kn strain of this study with already published sequences of KSGP O-240 and KS-1 strains provides further evidence that KSGP strains are not sufficiently attenuated in vaccines, causing clinical signs in previously unvaccinated naïve animals and, to confirm this statement, additional protection data and transmission studies would be important. If the strain is under-attenuated for cattle, it cannot be excluded that KSGP O-240 or O-180 strains may spread from vaccinated to unvaccinated naïve animals in the field setting.

It also needs to be considered that in real life, some vaccinated animals may already be incubating the virulent field strain when vaccinated. In this case, if the used vaccine is not sufficiently attenuated, it may result in a recombination of the genomes of the two viruses, i.e., the vaccine and the field virus, after entry into the same cell.

## 5. Conclusions

Results of this experiment highlight constraints related to the use of live vaccines, especially to protect against LSD. Under controlled conditions, groups of naïve healthy calves were vaccinated with two LSD vaccines based on Nt and Kn strains, at two different doses. Cases showing LSD-like lesions were observed among 69 vaccinated animals (8.7%). Lesions were observed more frequently when a high vaccine dose (12%) was used compared to the normal dose (37%). The percentage was higher in the Kn strain. Vaccination induced satisfactory seroconversion, confirming that the Nt strain can be used for mass vaccination to protect cattle against LSD. However, precaution should be taken not to use a high vaccine dose, which is likely to cause adverse reactions in vaccinated animals.

## Figures and Tables

**Table 1:** Scoring of recorded clinical signs.

Clinical Signs		Score
General behavior	Normal	0
	Inactive	1
	Very inactive	2
Fever	Normal	0
	39–40 °C	1
	>40 °C	2
Food uptake	Normal	0
	Loss of appetite	1
	Anorexia	2
Local inflammation	None	0
	<2 cm	1
	>2 cm	2
Neethling Disease	None	0
	1 to 2 nodules in one area	1
	Up to 5 nodules in one area	2
	>5 nodules in two areas	3
	>5 nodules in three areas	4
	Generalized nodules	5

**Table 2:** SNPs (single-nucleotide polymorphisms) of KSGP O-240 refreshed on animal.

Reference Position	Type	Length	Ref	Allele	Overlapping Annotations	Coding Region Change	Amino Acid Change
17	SNV	1	T	A			
18,578	Deletion	1	A	-	Gene: LSDV026, CDS: LSDV026	AOE47602.1:c.479del	AOE47602.1:p.Leu160fs
22,772	Insertion	1	-	A			
28,073	Insertion	1	-	T			
84,168	SNV	1	C	A	Gene: LSDV089, CDS: LSDV089	AOE47665.1:c.48G > T	AOE47665.1:p.Leu16Phe
89,076	SNV	1	G	T	Gene: LSDV094, CDS: LSDV094	AOE47670.1:c.254C > A	AOE47670.1:p.Pro85His
125,083	Deletion	1	A	-			

**Table 3:** Clinical scoring in animals vaccinated with Neethling t and Kenyan strains at different doses.

Group		G1	G2	G3	G4	G5
Vaccine		Neethling Strain		Kenyan Strain		Unvaccinated
Dose		Low	High	Low	High	-
Number of animals		15	30	12	12	4
Fever (days/animal)		0.93	1.87	2.33	2.92	0
Duration of fever in days		2	4.3	5.6	3.8	0
Number of animals showing local reaction at the inoculations site		0	2	0	1	0
Clinical score		1	1	1	2.5	0
Neethling disease	Number of cases	1	2	0	3	0
	Percentage	6.7%	6.7%	0%	25.0%	0%

**Table 4:** A total cumulative scoring of animals presenting LSD-like disease.

Group	Animal ID	Fever	Local Inflammation	Neethling Disease	Total Score
G1	833	1	0	1	2
G2	906	1	2	2	5
	4599	1	2	2	5
G3	All	1	0	0	1
G4	661	2	0	5	7
	5136	1	0	3	4
	698	2	2	4	8

**Table 5:** Percentage of positive animals by VNT.

Vaccine		A Total Number of Seropositive Animals/Day Post Vaccination								
Group	Number of Animals	D0	D7	D14	D21	D28	D35	D42	D60	D90
Nt low dose (G1)	15	0	0	3	6	6	6	7	7	6
Nt high dose (G2)	30	0	5	8	15	20	22	22	22	14
Kn low dose (G3)	12	0	0	8	12	12	12	12	12	9
Kn high dose (G4)	12	0	3	10	12	12	12	12	12	12
Unvaccinated (G5)	5	0	0	0	0	0	0	0	0	0

## Data Availability

The datasets used and/or analyzed during the current study are available from the corresponding author on reasonable request. All recorded raw data are archived in MCI Santé Animale.
